# QuasR: quantification and annotation of short reads in R

**DOI:** 10.1093/bioinformatics/btu781

**Published:** 2014-12-09

**Authors:** Dimos Gaidatzis, Anita Lerch, Florian Hahne, Michael B. Stadler

**Affiliations:** ^1^Friedrich Miescher Institute for Biomedical Research, Maulbeerstrasse 66, 4058 Basel, Switzerland, ^2^Swiss Institute of Bioinformatics, 4058 Basel, Switzerland and ^3^Novartis Institute for Biomedical Research, CH-4057 Basel, Switzerland

## Abstract

**Summary:** QuasR is a package for the integrated analysis of high-throughput sequencing data in R, covering all steps from read preprocessing, alignment and quality control to quantification. QuasR supports different experiment types (including RNA-seq, ChIP-seq and Bis-seq) and analysis variants (e.g. paired-end, stranded, spliced and allele-specific), and is integrated in Bioconductor so that its output can be directly processed for statistical analysis and visualization.

**Availability and implementation:** QuasR is implemented in R and C/C++. Source code and binaries for major platforms (Linux, OS X and MS Windows) are available from Bioconductor (www.bioconductor.org/packages/release/bioc/html/QuasR.html). The package includes a ‘vignette’ with step-by-step examples for typical work ﬂows.

**Contact:** michael.stadler@fmi.ch

**Supplementary information:** Supplementary data are available at *Bioinformatics* online.

## 1 Introduction

High-throughput sequencing has become a powerful research tool in a wide range of applications, such as transcriptome profiling (RNA-seq), measurement of DNA-protein interactions or chromatin modifications (ChIP-seq) and DNA methylation (Bis-seq). In the last years, there have been many efforts to provide software in R/Bioconductor (Gentleman *et al.*, 2004) to simplify data processing and biological interpretation, such as an efficient framework for working with genomic ranges (Lawrence *et al.*, 2013), or tools for read alignment (Liao *et al.*, 2013), quality control (Morgan *et al.*, 2009) and statistical analysis (Anders and Huber 2010; Robinson *et al.*, 2010). It is however still challenging to conduct a complete analysis from raw data to a publishable result in the form of a single R script, which would greatly improve documentation and thus facilitate the exchange of analysis details with coworkers. Often it is necessary to perform a subset of tasks outside of R, for example on the operating system’s shell. Typically, the tools used in the analysis have to be downloaded and installed from distinct sources, and resolving software dependencies can be time-consuming or become a major obstacle for non-expert researchers trying to perform or reproduce an analysis.

Here, we introduce the Bioconductor package QuasR that aims to overcome these issues by abstracting many technical details of high-throughput sequencing data analysis. QuasR builds on top of the functionality provided by Bioconductor and external tools such as bowtie (Langmead *et al.*, 2009) or SpliceMap (Au *et al.*, 2010), and extends it to support additional analysis types, such as DNA methylation and allele-specific analysis. QuasR is available for all major platforms (Linux, OS X and MS Windows), and its output can be directly used for downstream analyses and visualization, thus allowing an uninterrupted workflow from raw data to scientific results.

## 2 Feature overview

**Fig. 1.**
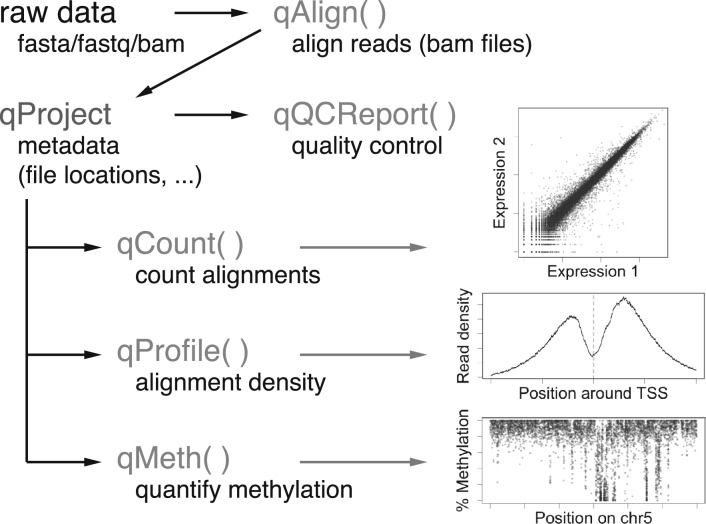
QuasR consists of one class (*qProject*) and five main functions. Typical visualizations of the function outputs are shown as insets

The user interface of QuasR consists of only a handful of functions (Fig. 1) and a single class (*qProject*) that is returned by *qAlign* and serves as input to all downstream processing.

By default, *qAlign* uses bowtie to align single or paired-end reads to a reference genome, and unmapped reads are optionally further aligned to alternative references, for example to quantify the level of vector contamination. For convenience, the genome can be obtained through Bioconductor (42 genome assemblies for 21 different species are available in release 2.14), in which case it is automatically downloaded and indexed if necessary. *qAlign* also supports spliced alignments and alignment of bisulfite-converted reads (both directional und undirectional bisulfite libraries). Pre-existing alignments in BAM format (Li *et al.*, 2009) that have been generated outside of QuasR can be imported, thereby enabling the use of any alignment software and strategy that produces output in the supported format. Finally, *qAlign* stores metadata for all generated BAM files, including information about alignment parameters and checksums for genome and short read sequences, allowing it to recognize pre-existing BAM files that will not have to be recreated.

*qQCReport* produces a set of quality control plots that allow assessment of the technical quality of sequencing data and alignments, and help to identify over-represented reads and libraries with a low sequence complexity.

*qCount* is the main function for quantification. It is used to count alignments in known genomic intervals (promoters, exons, genes, etc.) or in peak regions identified outside of QuasR. It avoids redundant counting of individual alignments (e.g. when combining transcripts from the same gene). *qCount* provides fine-grained control over quantification, for example to include only alignments that are (anti-)sense to the query region, to select alignments based on mapping quality or to report counts for exon–exon junctions. The resulting count tables can be directly used for statistical analysis in dedicated packages (Anders and Huber, 2010; Robinson *et al.*, 2010).

*qProfile* is similar to *qCount,* with the main difference that it returns a spatial profile of counts with the number of alignments at different positions relative to the query.

*qMeth* is used in Bis-seq experiments to obtain the numbers of methylated and unmethylated cytosines for selected sequence contexts.

For experimental systems with known heterozygous loci (for example an F1 cross between two divergent mouse inbred strains), QuasR allows to perform allele-specific analysis. In order to avoid alignment bias, *qAlign* will automatically inject the known single nucleotide variations into the reference genome to produce two new versions of that genome. The reads are aligned to both genomes, and the best alignment for each read is retained. The quantification of such allele-specific alignments by *qCount, qProfile* or *qMeth* produces three instead of a single number per sample and query feature, corresponding to the alignment counts for reference and alternative alleles and the unclassifiable alignments. An alignment can be unclassifiable if the read (or both reads in a paired-end experiment) did not overlap with a known polymorphism.

All QuasR functions are designed to make use of the parallel package for parallel processing on computers with multiple cores or compute clusters.

The package vignette (available at http://www.bioconductor.org/packages/release/bioc/vignettes/QuasR/inst/doc/QuasR.pdf) contains more details on QuasR functions, as well as step-by-step examples for typical analysis tasks. In addition, the supplementary online material provides QuasR code recipes illustrating the installation of the QuasR package, removal of adaptor sequences, quantification of RNA expression and DNA methylation, as well as allele-specific analysis.

## 3 Conclusions

By abstracting technical details, QuasR greatly simplifies an analysis of high-throughput sequencing data and makes it accessible to a wider community. Already the installation of required software tools can be conveniently achieved from within R, irrespective of the compute platform. Furthermore, through its integration with the Bioconductor infrastructure, it is also possible to obtain genome sequences and gene annotation in this manner, and the R packaging system provides a solid infrastructure to track and document the versions of both software and annotation that is used in a given analysis, which is a prerequisite for reproducible research.

QuasR unites the preprocessing and alignment of raw sequence reads with the numerous downstream analysis tools available in Bioconductor and for the R environment, and enables well integrated, single-script workflows that document all steps of an analysis and their parameters in a format that is simple to share and reproduce.

## Supplementary Material

Supplementary Data
