# Supplementary material for: QuasR: quantification and annotation of short reads in R
Source: Bioinformatics. 2014 Dec 9;31(7):1130–2. doi: 10.1093/bioinformatics/btu781 (PMC4382904; doi:10.1093/bioinformatics/btu781)
Supplement: Supplementary Data [file supp_31_7_1130__index.html]

QuasR: quantification and annotation of short reads in R — Supplementary Data 

# QuasR: quantification and annotation of short reads in R

## Supplementary Data

files

**Files in this Data Supplement:**

- Supplementary Data - pdf file
