# Supplementary material for: QuasR: quantification and annotation of short reads in R
Source: Bioinformatics. 2014 Dec 9;31(7):1130–2. doi: 10.1093/bioinformatics/btu781 (PMC4382904; doi:10.1093/bioinformatics/btu781)
Supplement: Supplementary Data [file supp_btu781_quasr_supp_final.pdf]

Supplementary Material:
QuasR: Quantification and annotation of short reads in R

Dimos Gaidatzis, Anita Lerch, Florian Hahne and Michael B. Stadler

Contents

Recipe 1: Download and install QuasR 1

Recipe 2: Copy sample data to a temporary directory 1

Recipe 3: Removing of adaptor sequences from raw reads 2

Recipe 4 (RNA-seq): Calculate RNA levels for differential expression analysis 2

Recipe 5 (ChIP-seq): Export `wig` files and create a metagene profile plot 5

Recipe 6 (Bis-seq): Calculate methylation states 7

Recipe 7: Allele-specific analysis 9

Appendix A: QuasR hardware requirements and running time 10

Appendix B: Session info and package versions 12

References 13

Recipe 1: Download and install QuasR

The following code will download and install the current version of **QuasR** from **Bioconductor** (see also `http://www.bioconductor.org/packages/release/bioc/html/QuasR.html`):

```
source("http://bioconductor.org/biocLite.R")
biocLite("QuasR")
```

In addition to **QuasR**, this may also download and install additional packages that it depends on. After the installation has finished, the library is loaded to make its functionality available:

```
library(QuasR)
```

Recipe 2: Copy sample data to a temporary directory

The following command copies the `extdata` folder that comes with **QuasR** into your current working directory. This folder contains sample data that will be used in the recipes below.

```r
file.copy(system.file(package="QuasR", "extdata"), ".", recursive=TRUE)
```

Recipe 3: Removing of adaptor sequences from raw reads

In this recipe, *preprocessReads* is used to process the raw sequence files in `infiles` and store the processed sequences in `outfiles`. In the example here the processing consists of truncating the last three bases, removing poly-adenine from the start and filtering out all reads that are shorter than 14 bases (after truncation and trimming) or contain two or more undetermined nucleotides (`nBases`). As illustrated below, input and output files can be compressed. *preprocessReads* returns a matrix with the number of processed sequences for each file.

```r
infiles <- file.path("extdata",c("rna_1_1.fq.bz2","rna_2_1.fq.bz2"))
outfiles <- file.path("extdata",paste("processed_",basename(infiles),sep=""))
res <- preprocessReads(filename = infiles,
                       outputFilename = outfiles,
                       truncateEndBases = 3,
                       Lpattern = "AAAAAAAAAA",
                       minLength = 14,
                       nBases = 2)

##  filtering extdata/rna_1_1.fq.bz2
##  filtering extdata/rna_2_1.fq.bz2

res

##                  rna_1_1.fq.bz2 rna_2_1.fq.bz2
## totalSequences             3002           3000
## matchTo5pAdapter            466            463
## matchTo3pAdapter              0              0
## tooShort                    107             91
## tooManyN                      0              0
## lowComplexity                 0              0
## totalPassed                2895           2909
```

Recipe 4 (RNA-seq): Calculate RNA levels for differential expression analysis

In this recipe, *qCount* is used to count alignments per gene and sample, generating a count table suitable for identification of differentially expressed genes. It begins by creating spliced alignments to the reference genome (here a fasta formatted file) using *qAlign*. Alternatively, the genome could be specified using the name of a `BSgenome` package (e.g. `"BSgenome.Hsapiens.UCSC.hg19"`), which will be automatically downloaded and indexed if necessary.

```r
# create alignments
sampleFile <- "extdata/samples_rna_single.txt"
genomeFile <- "extdata/hg19sub.fa"
proj <- qAlign(sampleFile, genome=genomeFile, splicedAlignment=TRUE)

## Creating .fai file for:  extdata/hg19sub.fa
## alignment files missing - need to:
##     create alignment index for the genome
```

```
##    create 4 genomic alignment(s)
## Creating an Rbowtie index for extdata/hg19sub.fa
## Finished creating index
## Testing the compute nodes...OK
## Loading QuasR on the compute nodes...OK
## Available cores:

## nodeNames
##        computenode
##                  1

## Performing genomic alignments for 4 samples.  See progress in the log file:
## QuasR_log_5415792cc45.txt
## Genomic alignments have been created successfully

proj

## Project: qProject
##  Options   : maxHits         : 1
##              paired          : no
##              splicedAlignment: TRUE
##              bisulfite       : no
##              snpFile         : none
##  Aligner   : Rbowtie v1.4.5 (parameters: -max_intron 400000...)
##  Genome    : hg19sub.fa (file)
##
##  Reads     : 4 files, 2 samples (fastq format):
##    1. rna_1_1.fq.bz2  Sample1 (phred33)
##    2. rna_1_2.fq.bz2  Sample1 (phred33)
##    3. rna_2_1.fq.bz2  Sample2 (phred33)
##    4. rna_2_2.fq.bz2  Sample2 (phred33)
##
##  Genome alignments: directory: same as reads
##    1. rna_1_1_541536a0c9e5.bam
##    2. rna_1_2_54157626f567.bam
##    3. rna_2_1_541577b979ca.bam
##    4. rna_2_2_541531eafa2e.bam
##
##  Aux. alignments: none
```

For the definition of gene models, a `GRanges` object is constructed containing exon ranges named by gene. Multiple ranges (exons) with the same name will be automatically combined by *qCount* in a non-redundant manner, assuring that a single read is not counted multiple times. As an alternative, a `TranscriptDb` object can be used to define gene models for *qCount* (see **QuasR** vignette for examples).

```
# read in gene annotation
library(rtracklayer)
gr <- import("extdata/hg19sub_annotation.gtf")
gr <- gr[gr$type=="exon"]
names(gr) <- gr$gene_name
```

The third step is the call to *qCount* that counts for each gene the alignments overlapping with any of its exons. The resulting count table also contains the cumulative length for each feature or combination of features (here: the cumulative, non-redundant number of exonic bases in any transcript of that gene), making it simple to scale the counts by length and sequencing depth and to calculate for example RPKM (or FPKM) values or similar.

```r
# quantify expression
cnt <- qCount(proj, gr)

## counting alignments...done
## collapsing counts by sample...done
## collapsing counts by query name...done

head(cnt)

##             width Sample1 Sample2
## TNFRSF18     1370      26       2
## TNFRSF4      1721      37       8
## SDF4         4697     705    1078
## B3GALT6      2793      62     344
## AC108488.1   1104       0       0
## RPS7         5583    2922    2224

# calculate RPKM
geneRPKM <- t(t(cnt[,-1] /cnt[,1] *1000) /colSums(cnt[,-1]) *1e6)
head(geneRPKM)

##             Sample1 Sample2
## TNFRSF18       2692   201.6
## TNFRSF4        3049   642.0
## SDF4          21287 31695.6
## B3GALT6        3148 17009.4
## AC108488.1        0     0.0
## RPS7          74227 55013.4
```

Please note the RPKM values in our example are higher than what you would usually get for a real RNA-seq dataset. The values here are artificially scaled up because our example data contains reads only for a small number of genes.

The count table returned by *qCount* is also the basis for downstream statistical analysis using packages such as `edgeR` (Robinson et al., 2010), `DESeq` (Anders and Huber, 2010), `DESeq2` (Love et al., 2014), `TCC` (Sun et al., 2013), `DEXSeq` (Anders et al., 2012) or `baySeq` (Hardcastle and Kelly, 2010). The code below shows how it can be converted into a `edgeR::DEGList` or a `DESeq2::DESeqDataSet` object, although in this case no replicates are available and thus the example data is not well suited for a statistical analysis.

```r
# prepare experiment information
geneinfo <- data.frame(identifier=rownames(cnt), width=cnt[,1])
group <- factor(c("wt","ko"))
sampleinfo <- data.frame(sampleName=colnames(cnt)[-1], group=group)

# make a DGEList for edgeR
library(edgeR)
d <- DGEList(counts=cnt[,-1], group=group, genes=geneinfo)

# make a DESeqDataSet for DESeq2
library(DESeq2)
dds <- DESeqDataSetFromMatrix(countData=cnt[,-1], colData=sampleinfo, design= ~ group)
```

Recipe 5 (ChIP-seq): Export wig files and create a metagene profile plot

In this recipe, reads from a ChIP-seq experiment are aligned, and the alignment density along the genome is exported in wig format for visualization in public genome browsers.

```
# create alignments
sampleFile <- "extdata/samples_chip_single.txt"
genomeFile <- "extdata/hg19sub.fa"
proj <- qAlign(sampleFile, genome=genomeFile)

## alignment files missing - need to:
##     create 2 genomic alignment(s)
## Testing the compute nodes...OK
## Loading QuasR on the compute nodes...OK
## Available cores:

## nodeNames
##        computenode
##                  1

## Performing genomic alignments for 2 samples.  See progress in the log file:
## QuasR_log_529c441257ef.txt
## Genomic alignments have been created successfully

proj

## Project: qProject
##  Options   : maxHits         : 1
##              paired          : no
##              splicedAlignment: FALSE
##              bisulfite       : no
##              snpFile         : none
##  Aligner   : Rbowtie v1.4.5 (parameters: -m 1 --best --strata)
##  Genome    : hg19sub.fa (file)
##
##  Reads     : 2 files, 2 samples (fastq format):
##    1. chip_1_1.fq.bz2  Sample1 (phred33)
##    2. chip_2_1.fq.bz2  Sample2 (phred33)
##
##  Genome alignments: directory: same as reads
##    1. chip_1_1_529c104b2ed3.bam
##    2. chip_2_1_529c37ef2700.bam
##
##  Aux. alignments: none

# export alignment density to wig files
qExportWig(proj, binsize=100, scaling=TRUE)

## collecting mapping statistics for scaling...done
## start creating wig file(s)...
##  Sample1.wig.gz (Sample1)
##  Sample2.wig.gz (Sample2)
## done
```

Next, the alignment density around transcript start sites (TSS) is plotted, separately for sense and antisense alignments to create a metagene profile. This plot reveals the average ChIP fragment size as a shift between the sense and antisense alignment densities.

```r
# read in gene annotation
library(rtracklayer)
gr <- import("extdata/hg19sub_annotation.gtf")

# get TSS coordinates
grFirstExon <- gr[gr$type=="exon" & gr$exon_number==1]
grFirstBase <- resize(grFirstExon,width=1,fix="start")
grTSS <- unique(grFirstBase)

# quantify alignments around TSS (separately for each strand)
prS <- qProfile(proj, grTSS, upstream=3000, downstream=3000, orientation="same")

## profiling alignments...done

prO <- qProfile(proj, grTSS, upstream=3000, downstream=3000, orientation="opposite")

## profiling alignments...done

# combine data from all TSS
prCombS <- do.call("+", prS[-1]) /prS[[1]]
prCombO <- do.call("+", prO[-1]) /prO[[1]]

# metagene profile plot
prCombSsmooth <- filter(prCombS[1,], rep(1/100,100))
prCombOsmooth <- filter(prCombO[1,], rep(1/100,100))
plot(as.numeric(colnames(prCombS)), prCombSsmooth, type="l",
     xlab="Position relative to TSS", ylab="Mean no. of alignments",
     ylim=c(0,max(prCombSsmooth,prCombOsmooth,na.rm=TRUE)))
lines(as.numeric(colnames(prCombO)), prCombOsmooth, col="red")
legend(title="strand", legend=c("same as query","opposite of query"),
       x="topleft", col=c("black","red"), lwd=1.5, bty="n", title.adj=0.1)
```

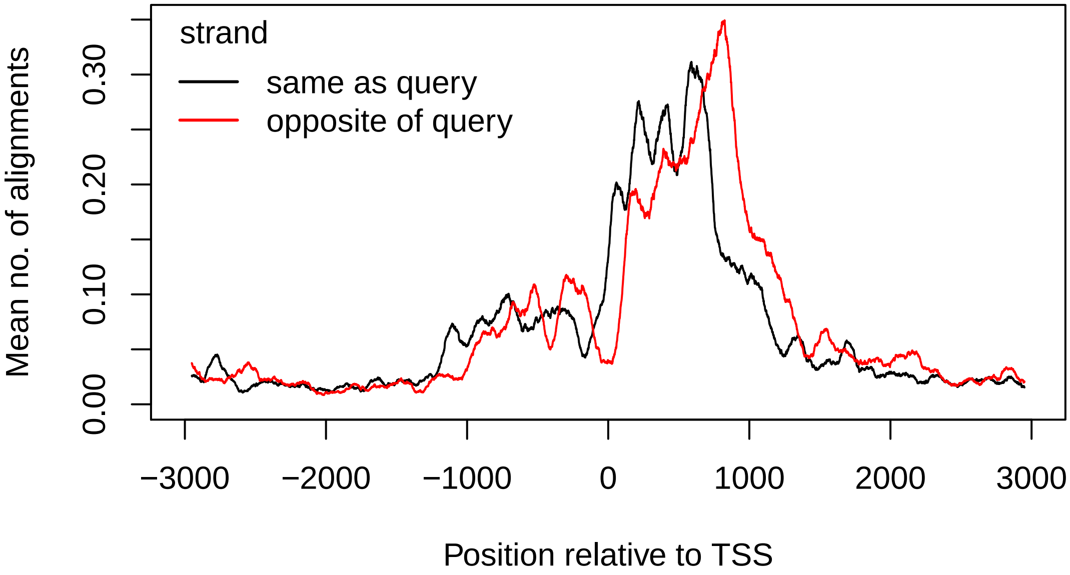

Recipe 6 (Bis-seq): Calculate methylation states

The methylation of cytosines can be analyzed with **QuasR** using bisulphite sequencing (Bis-seq) data. **QuasR** supports both directed and undirected Bis-seq libraries, as specified using the `bisulfite` parameter. If this parameter is set, *qAlign* will bisulphite-convert both reads and genome and align the reads with a three-letter alphabet. The *in silico*-converted cytosines are then put back into the resulting alignments, and methylation states are quantified using *qMeth*. The resulting output can for example be used to identify partially methylated domains (PMDs) and regions of low methylation (LMRs and UMRs) using the MethylSeekR package (Burger et al., 2013).

```
# create Bis-seq alignments
sampleFile <- "extdata/samples_bis_single.txt"
genomeFile <- "extdata/hg19sub.fa"
proj <- qAlign(sampleFile, genomeFile, bisulfite="dir")

## alignment files missing - need to:
##    create alignment index for the genome
##    create 1 genomic alignment(s)
## Creating an RbowtieCtoT index for extdata/hg19sub.fa
## Finished creating index
## Testing the compute nodes...OK
## Loading QuasR on the compute nodes...OK
## Available cores:

## nodeNames
##        computenode
##                  1
```

```
## Performing genomic alignments for 1 samples.  See progress in the log file:
## QuasR_log_529c504bc758.txt
## Genomic alignments have been created successfully

# quantify CpG methylation
meth <- qMeth(proj, mode="CpGcomb", collapseBySample=TRUE)
meth

## GRanges with 3110 ranges and 2 metadata columns:
##          seqnames         ranges strand   | Sample1_T Sample1_M
##             <Rle>      <IRanges>  <Rle>   | <integer> <integer>
##      [1]     chr1       [19, 20]      *   |         1         1
##      [2]     chr1       [21, 22]      *   |         1         1
##      [3]     chr1       [54, 55]      *   |         3         1
##      [4]     chr1       [57, 58]      *   |         3         0
##      [5]     chr1       [80, 81]      *   |         6         5
##      ...      ...            ...    ... ...       ...       ...
##   [3106]     chr3 [44957, 44958]      *   |         8         7
##   [3107]     chr3 [44977, 44978]      *   |         5         3
##   [3108]     chr3 [44981, 44982]      *   |         4         3
##   [3109]     chr3 [44989, 44990]      *   |         1         1
##   [3110]     chr3 [44993, 44994]      *   |         1         1
##   ---
##   seqlengths:
##     chr1  chr2  chr3
##    40000 10000 45000

# visualize (chr3 contains two CpG islands)
percMeth <- mcols(meth)[,2] *100 /mcols(meth)[,1]
i <- as.logical(seqnames(meth)=="chr3")
plot(start(meth)[i], percMeth[i], pch=19, col="#22222266",
     xlab="Position on chr3 (bp)", ylab="CpG methylation")
```

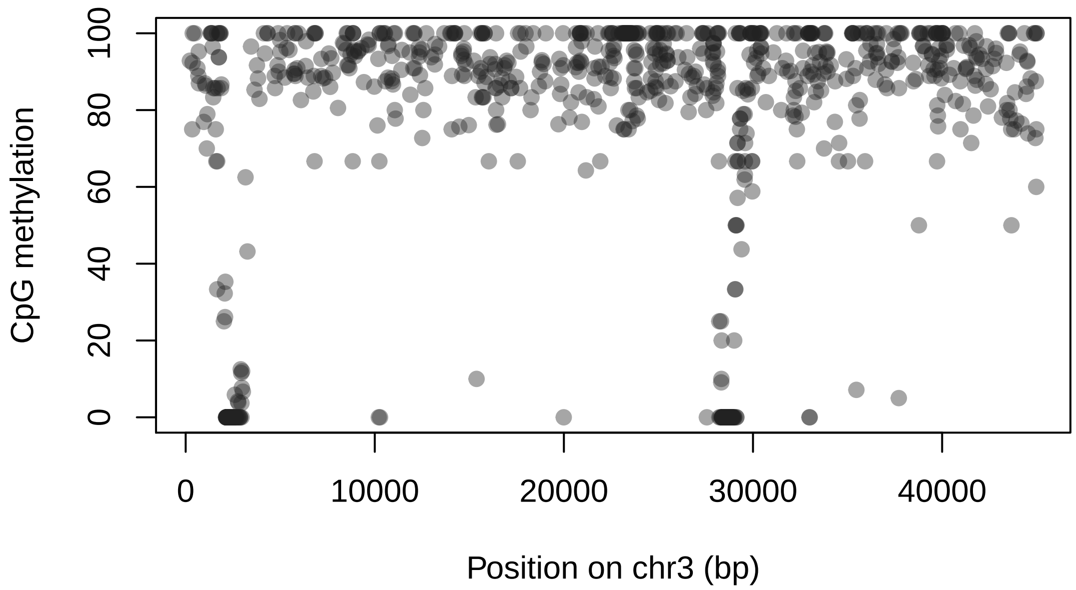

Recipe 7: Allele-specific analysis

All experiment types supported by **QuasR** (ChIP-seq, RNA-seq and Bis-seq; only alignments to the genome, but not to auxiliaries) can also be analyzed in an allele-specific manner. For this, a file containing genomic location and the two alleles of known sequence polymorphisms has to be provided to *qAlign*. The recipie uses the file available from `system.file(package="QuasR", "extdata", "hg19sub_snp.txt")`. To avoid an alignment bias, all reads are aligned separately to each of the two new genomes, which **QuasR** generates by injecting the polymorphisms listed in `snpFile` into the reference genome, only retaining the best alignment for each read. While combining alignments from the two genomes into the final `BAM` file, each read is classified into one of three groups:

- **R**: the read aligned better to the reference genome
- **U**: the read aligned equally well to both genomes (unknown origin)
- **A**: the read aligned better to the alternative genome

Using this alignment classification, the *qCount*, *qProfile* and *qMeth* functions will produce three counts instead of a single count for each feature. The column names are suffixed by `_R`, `_U` and `_A`.

```
# create alignments
sampleFile <- "extdata/samples_chip_single.txt"
genomeFile <- "extdata/hg19sub.fa"
snpFile <- "extdata/hg19sub_snp.txt"
proj <- qAlign(sampleFile, genome=genomeFile, snpFile=snpFile)

## alignment files missing - need to:
##     create alignment index for the genome
##     create 2 genomic alignment(s)
## Reading and processing the SNP file:  extdata/hg19sub_snp.txt
```

```
## Creating the genome fasta file containing the SNPs:  extdata/hg19sub_snp.txt.hg19sub.fa.R.fa
## Creating the genome fasta file containing the SNPs:  extdata/hg19sub_snp.txt.hg19sub.fa.A.fa
## Creating a .fai file for the snp genome:  extdata/hg19sub_snp.txt.hg19sub.fa.R.fa
## Creating a .fai file for the snp genome:  extdata/hg19sub_snp.txt.hg19sub.fa.A.fa
## Creating an Rbowtie index for extdata/hg19sub_snp.txt.hg19sub.fa.R.fa
## Finished creating index
## Creating an Rbowtie index for extdata/hg19sub_snp.txt.hg19sub.fa.A.fa
## Finished creating index
## Testing the compute nodes...OK
## Loading QuasR on the compute nodes...OK
## Available cores:

## nodeNames
##        computenode
##                  1

## Performing genomic alignments for 2 samples.  See progress in the log file:
## QuasR_log_529c249a94b7.txt
## Genomic alignments have been created successfully

# get promoter regions from annotation
library(rtracklayer)
gr <- import("extdata/hg19sub_annotation.gtf")
names(gr) <- gr$transcript_id
grFirstExon <- gr[gr$type=="exon" & gr$exon_number==1]
grPromoter <- flank(unique(grFirstExon),0)+500

# allele-specific quantification
cnt <- qCount(proj, grPromoter)

## counting alignments...done

cnt[1:6,1:4]

##                 width Sample1_R Sample1_U Sample1_A
## ENST00000486728  1000         3        12         0
## ENST00000328596  1000         4        14         0
## ENST00000379268  1000         4        14         0
## ENST00000453580  1000         0         7         0
## ENST00000379236  1000         0         5         0
## ENST00000497869  1000         0         5         0
```

Appendix A: QuasR hardware requirements and running time

Minimal hardware requirements

QuasR was designed to have low minimal hardware requirements, but to use multiple CPU cores if they are available. It has been tested on a laptop computer with 4Gb of memory and a dual-core CPU. The minimally required memory is mainly determined by the requirements of the alignment tool. By default, this is `bowtie` which requires about 2.2Gb of memory for alignments to human or mouse genomes (2.9Gb for paired-end experiments).

Hardware and data used in performance measurements

The **QuasR** running times reported below were obtained on an IBM® x3850 X5 computer with Intel® Xeon® X7542 CPUs with a clock rate of 2.67GHz. Reported times correspond to elapsed time as measured by `system.time` (the minimum over three independent trials) and were obtained on three samples selected from an RNA-seq dataset that is publicly available from GEO (`http://www.ncbi.nlm.nih.gov/geo/query/acc.cgi?acc=GSE33252`) under the series accession GSE33252 (Tippmann et al., 2012). In total, the three samples contain about 100 Mio. reads:

Sample name	GEO Identifier	No. reads	No. alignments
ES_RNA_a	GSM778487	38.5 Mio.	16.7 Mio.
NP_RNA_a	GSM778489	33.8 Mio.	13.4 Mio.
TN_RNA_a	GSM778491	30.4 Mio.	11.8 Mio.

Typical `qAlign` running time

We first measured the time to generate alignments with `qAlign` against the mouse genome (`BSgenome` package pre-indexed with **QuasR**) using default parameters and different numbers of CPU cores (`n`):

```
cl <- makeCluster(n)
proj <- qAlign("samples.txt", "BSgenome.Mmusculus.UCSC.mm10", clObj=cl)
```

The following figure shows the observed execution times as a function of the number of CPU cores (left panel, logarithmic axes) and the resulting speedup (ratio of serial over parallel time, right panel). The ∼ 100 Mio. reads are aligned in about 5 hours on a single CPU, and that time linearly decreases up to about four cores. Starting with eight cores, the speedup is sub-linear due to sequential parts in the alignment generation process with contribute a constant amount of execution time.

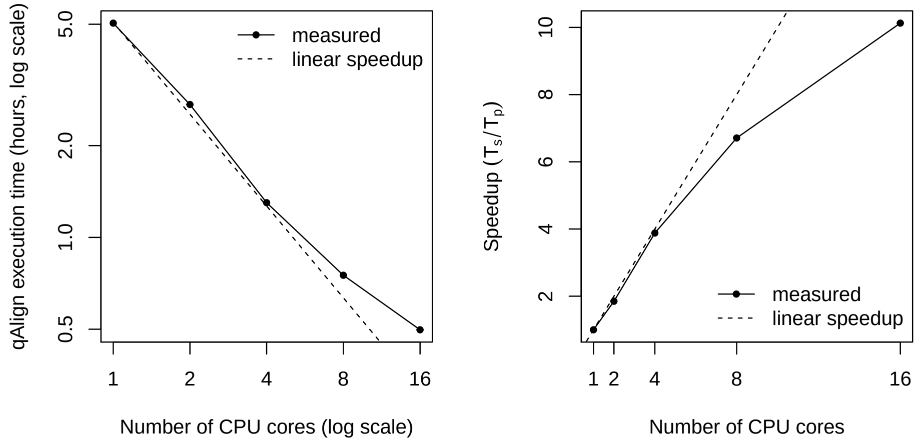

Typical `qCount` running time

Next, we measured execution time of counting alignments for each gene with `qCount`. We first created a `GRanges` query object `gr` extracted from the `TxDb.Mmusculus.UCSC.mm10.knownGene` package, which contained a total of 218,671 exons for 23,725 genes. This object was used as a query in `qCount` to count alignments in all three samples using:

```
qCount(proj, gr, clObj=cl)
```

The figure below shows `qCount` execution times (left) and speedup (right) for alignment counting. For `qCount`, the speedup starts to become sub-linear already with four cores, which may be due to the larger

fraction of serial code in `qCount` compared to `qAlign`. Furthermore, the performance of the storage system when accessing the alignment files can also limit the speedup.

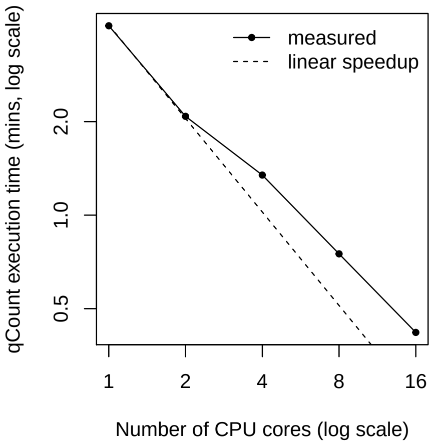

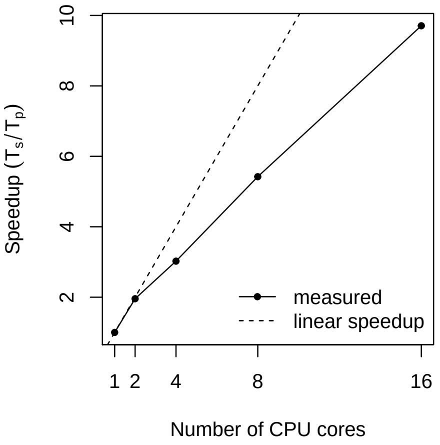

QuasR performance summary

The table below contains a summary of all benchmark results from above (time in seconds):

n_{cores}	$T_{alignment}$	$Speedup_{alignment}$	T_{count}	$Speedup_{count}$
1	18153	1.00	244	1.00
2	9819	1.85	125	1.96
4	4679	3.88	81	3.02
8	2706	6.71	45	5.42
16	1792	10.13	25	9.71

Appendix B: Session info and package versions

```
sessionInfo()

## R version 3.1.1 (2014-07-10)
## Platform: x86_64-apple-darwin13.1.0 (64-bit)
##
## locale:
## [1] en_US.UTF-8/en_US.UTF-8/en_US.UTF-8/C/en_US.UTF-8/en_US.UTF-8
##
## attached base packages:
## [1] stats4    parallel  stats     graphics  grDevices utils     datasets
## [8] methods   base
##
## other attached packages:
## [1] QuasR_1.5.4           Rbowtie_1.5.5         GenomicRanges_1.17.48
## [4] GenomeInfoDb_1.1.26   IRanges_1.99.32       S4Vectors_0.2.8
## [7] BiocGenerics_0.11.5   knitr_1.6
##
## loaded via a namespace (and not attached):
##  [1] AnnotationDbi_1.27.19    base64enc_0.1-2
##  [3] BatchJobs_1.4            BBmisc_1.7
##  [5] Biobase_2.25.1           BiocInstaller_1.15.5
```

```
##  [7] BiocParallel_0.99.27     biomaRt_2.21.5
##  [9] Biostrings_2.33.15       bitops_1.0-6
## [11] brew_1.0-6               BSgenome_1.33.9
## [13] checkmate_1.4            codetools_0.2-9
## [15] DBI_0.3.1                digest_0.6.4
## [17] evaluate_0.5.5           fail_1.2
## [19] foreach_1.4.2            formatR_1.0
## [21] GenomicAlignments_1.1.30 GenomicFeatures_1.17.22
## [23] grid_3.1.1               highr_0.3
## [25] hwriter_1.3.2            iterators_1.0.7
## [27] lattice_0.20-29          latticeExtra_0.6-26
## [29] RColorBrewer_1.0-5       RCurl_1.95-4.3
## [31] Rsamtools_1.17.36        RSQLite_0.11.4
## [33] rtracklayer_1.25.20      sendmailR_1.2-1
## [35] ShortRead_1.23.17        stringr_0.6.2
## [37] tools_3.1.1              XML_3.98-1.1
## [39] XVector_0.5.8            zlibbioc_1.11.1
```

References

Mark D Robinson, Davis J McCarthy, and Gordon K Smyth. edgeR: a bioconductor package for differential expression analysis of digital gene expression data. *Bioinformatics*, 26:139–140, 2010.

Simon Anders and Wolfgang Huber. Differential expression analysis for sequence count data. *Genome Biology*, 11:R106, 2010. doi: 10.1186/gb-2010-11-10-r106. URL http://genomebiology.com/2010/11/10/R106/.

Michael I Love, Wolfgang Huber, and Simon Anders. Moderated estimation of fold change and dispersion for RNA-Seq data with DESeq2. *bioRxiv*, 2014. doi: 10.1101/002832. URL http://dx.doi.org/10.1101/002832.

Jianqiang Sun, Tomoaki Nishiyama, Kentaro Shimizu1, and Koji Kadota. TCC: an R package for comparing tag count data with robust normalization strategies. *BMC Bioinformatics*, 14:219, 2013. doi: 10.1186/1471-2105-14-219. URL http://www.biomedcentral.com/1471-2105/14/219.

Simon Anders, Alejandro Reyes, and Wolfgang Huber. Detecting differential usage of exons from RNA-seq data. *Genome Research*, 22:2008–2017, 2012. doi: 10.1101/gr.133744.111. URL http://genome.cshlp.org/content/early/2012/06/21/gr.133744.111.full.pdf+html.

Thomas J Hardcastle and Krystyna A Kelly. baySeq: empirical bayesian methods for identifying differential expression in sequence count data. *BMC Bioinformatics*, 11:422, 2010. doi: 10.1186/1471-2105-11-422. URL http://www.biomedcentral.com/1471-2105/11/422.

Lukas Burger, Dimos Gaidatzis, Dirk Schuebeler, and Michael B. Stadler. Identification of active regulatory regions from DNA methylation data. *Nucleic Acids Research*, 41:e155, 2013. doi: 10.1093/nar/gkt599. URL http://nar.oxfordjournals.org/content/41/16/e155.full.

Sylvia C. Tippmann, Robert Ivanek, Dimos Gaidatzis, Anne Schoeler, Leslie Hoerner, Erik van Nimwegen, Peter F. Stadler, Michael B. Stadler, and Dirk Schuebeler. Chromatin measurements reveal contributions of synthesis and decay to steady-state mRNA levels. *Mol Syst Biol*, 8:593, 2012. doi: 10.1038/msb.2012.23. URL http://msb.embopress.org/content/8/1/593.long.
